# Early developments toward HbA_1c_ determination in whole blood by high-speed sample preparation and LC–MS/MS analysis

**DOI:** 10.1007/s00216-024-05601-5

**Published:** 2024-10-26

**Authors:** Indranil Mitra, Andreas Leinenbach, Andrea Geistanger, Andreas Huber, Thomas Dülffer, Susanne Adam, Lars Hillringhaus, Martin Silvestre, Holger Busskamp, Sven Vopel

**Affiliations:** Roche Diagnostics GmbH, Nonnenwald 2, 82377 Penzberg, Germany

**Keywords:** HbA_1c_, High-throughput, LC–MS/MS, Protein quantification, Clinical LC–MS

## Abstract

We report a method to determine HbA_1c_ (glycated hemoglobin) where whole blood samples are prepared by fast hemolysis (dilution with deionized water and vortex mixing), digestion with 0.6 mg/mL endoproteinase Glu C (Glu C) in 30 mM ammonium acetate buffer (pH 4.3) at 37 °C for 45 min, and termination of the digestion by diluting with 0.1% formic acid in water, and then analysis by a gradient liquid chromatography-tandem mass spectrometry (LC–MS/MS) method with a run time of 36 s. The method is linear between 0 and 200 HbA_1c_/mol Hb (IFCC) with a correlation coefficient of 0.999, providing an inter-day reproducibility between 1.3 and 2.3% CV, and comparable with results from analysis of the same samples on the Roche Cobas® c 513 clinical analyzer with a correlation coefficient of 0.998. In two alternative detection workflows that were not characterized in detail, the same digested samples were purified by a magnetic bead-based solid-phase extraction (SPE) method requiring about 10 min and then analyzed using either an isocratic LC–MS/MS method or a flow injection analysis (FIA)-MS/MS method with run times of 12 s and 18 s, respectively. Our work demonstrates the feasibility of LC–MS-based methods for HbA_1c_ determination that minimize the time required for sample preparation and measurement while preserving analytical performance and are thereby more suitable for routine clinical settings compared to traditional methods which require up to 25 h and 23 min, respectively, to prepare and measure samples.

**Graphical abstract:**

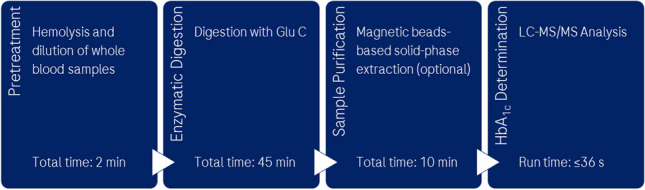

**Supplementary Information:**

The online version contains supplementary material available at 10.1007/s00216-024-05601-5.

## Introduction

HbA_1c_ (glycated hemoglobin) is globally accepted as the gold standard for diabetes testing [1–3]. In the blood stream, there is a fast and reversible equilibrium between free glucose and its reaction with the N-terminal valine of hemoglobin (Hb) and a slow formation of stable HbA_1c_. Accordingly, HbA_1c_ indicates the average glucose level in blood during the 2–3 months (average lifetime of red blood cells) prior to HbA_1c_ determination and is independent of the faster fluctuations in blood glucose levels. Although several different analytical techniques are routinely used for HbA_1c_ determination, mass spectrometry preserves accuracy for patient samples containing genetic Hb variants and other interferences by direct and simultaneous measurement of HbA_1c_ and HbA_0_ (non-glycated hemoglobin) and calibrating based on the ratio of these two signals [4–7].

A reference method approved by the International Federation of Clinical Chemistry and Laboratory Medicine (IFCC) determines HbA_1c_ from whole blood samples by proteolytic digestion of Hb with endoproteinase Glu C (Glu C) and targeted measurement of the resulting glycated and non-glycated N-terminal hexapeptides of the β-chain by liquid chromatography-mass spectrometry (LC–MS) [8–10]. The IFCC reference method provides an intra-laboratory reproducibility of ≤ 2% CV. Several methods have improved the speed and accuracy of the LC–MS detection but still require up to 25 h to prepare samples [11–18].

HbA_1c_ has also been determined by MS-based detection of intact Hb from whole blood. These strategies have focused on simple and fast sample preparation strategies (e.g., mild denaturation to dissociate the α-chains and β-chains of Hb and removal of salts and cellular debris by methods using solid supports, filtration, and dilution) and subsequent fast MS analysis by direct infusion, matrix-assisted laser desorption (MALDI), and capillary electrophoresis (CE) [6, 7, 19–25]. Although these methods are promising, LC–MS is advantageous for routine clinical HbA_1c_ diagnosis due to its ruggedness, reproducibility, versatility, and maturity.

In this work, we addressed the demand for MS-based determination of HbA_1c_ by reliable and reproducible methods that minimize the time required for sample preparation and measurement to be more suitable for routine clinical settings. Pretreatment and enzymatic digestion of whole blood samples were reduced to under 50 min by optimizing methods and conditions for fast hemolysis and Glu C digestion. A magnetic bead-based solid-phase extraction (SPE) method requiring less than 10 min was developed to improve sample purity. A gradient elution LC method with a run time of 36 s was developed on a Waters 2.1 × 50 mm LC column, and an isocratic elution LC method with a run time of 12 s was developed on a Waters 2.1 × 10 mm guard column. Moreover, a flow injection analysis (FIA) method was developed that used only a Waters in-line filter and had a run time of 18 s. The isocratic LC method with a run time of 12 s and the FIA method with a run time of 18 s were used in conjunction with the magnetic bead-based SPE workflow whereas the gradient LC method with a run time of 36 s did not require this additional sample purification step. All methods used a Thermo Vanquish HPLC system and performed targeted detection using positive-mode ESI and selected reaction monitoring (SRM) using a Thermo TSQ Quantiva triple quadrupole MS.

## Materials and methods

### Materials

The following materials were used: Ammonium acetate from Merck (101116); Acetic acid (glacial) from Merck (100063); D( +)-Glucose from Merck (8337); Sodium chloride from Merck (106404); Acetonitrile LC/MS grade from Biosolve (012078); Water LC/MS grade from Biosolve (232178); Formic acid ULC/MS grade from Biosolve (069141); Endoproteinase Glu C from Roche (10791156001); whole blood donated from participants at Roche, Penzberg; Magnetic beads for SPE from Roche; Waters XBridge Peptide BEH C18 Column, 300 Å, 3.5 um, 2.1 mm × 150 mm (186003609); Waters BEH C18 300 Å, 3.5 um, 2.1 × 50 mm (186003607); Waters Symmetry C18 Sentry Guard Cartridge 300 Å, 3.5 um, 2.1 × 10 mm (186000198); and Waters In-Line Filter Assembly (WAT035190). Calibrators were prepared manually from purified glycated and non-glycated Hb produced in-house at Roche Diagnostics GmbH, Penzberg. These materials are typically used to prepare candidate primary reference material approved by the IFCC. For details, please refer to Supplementary Table 1.

### Sample preparation

Table 1 compares the IFCC reference method and the method developed in this work for HbA_1c_ determination. For both workflows, 50 mM ammonium acetate buffer (pH 4.3) is prepared freshly on the day of experiments by dissolving 193 mg ammonium acetate in H_2_O, adjusting to pH 4.3 with 100% acetic acid, and filling up to 50 mL total.

**Table 1:** Comparison of the IFCC reference method to the method developed in this work for HbA_1c_ determination

Method steps	IFCC reference method	Method developed in this work
Pretreatment	Erythrocytes (red blood cells) are washed and extracted from whole blood samples (30 min) and incubated to destroy labile HbA_1c_ (4 h) using a saline solution Hemoglobin (Hb) is released by hemolysis of the purified erythrocytes and adjusted to a concentration of 50 mg/mL by dilution in a storage buffer (30 min) Total time: 5 h	Direct hemolysis and dilution of whole blood samples with deionized water Total time: 2 min
Enzymatic digestion	Samples are digested with 0.02 mg/mL Glu C in 50 mM ammonium acetate buffer (pH 4.3) at 37 °C The digestion is terminated by freezing the samples at − 20 °C Total time: 18–20 h	Samples are digested with 0.6 mg/mL Glu C in 30 mM ammonium acetate buffer (pH 4.3) at 37 °C The digestion is terminated by diluting the samples in 0.1% formic acid in water Total time: 45 min or 55 min
Sample purification	n.a	Solid-phase enrichment using magnetic beads provide a higher sample purity for the isocratic LC method and flow injection analysis whereas the gradient LC method did not require this additional sample purification step (see below) Total time: 10 min
Analysis	Targeted detection using positive-mode ESI and selected ion monitoring (SIM) LC using a 150-mm-long LC column and flow rate of 300 µL/min with gradient elution Run time: 23 min	Targeted detection using positive-mode ESI and selected reaction monitoring (SRM) LC using a 50-mm-long LC column and flow rate of 900 µL/min with gradient elution (Run time: 36 s) LC using a 10-mm-long pre-column and flow rate of 900 µL/min with isocratic elution (Run time: 12 s) Flow injection analysis using only an in-line filter and flow rate of 150 µL/min (Run time: 18 s)

The IFCC reference method requires up to 25 h for sample preparation, as detailed in Table 1. In contrast, the method developed and optimized in this work performs direct hemolysis and dilution of 100 µL whole blood samples with 900 µL deionized water by vortex mixing for 10 s. The hemolyzed whole blood sample (5 µL) is further diluted with 20 µL water. Glu C (2 mg/mL) is prepared freshly on the day of experiments by dissolving an entire 2-mg bottle in 1 mL water. Digestion of 5 µL of the hemolyzed and diluted samples with 0.6 mg/mL Glu C (15 µL added from 2 mg/mL Glu C) is done by incubation in 30 mM ammonium acetate buffer (30 µL added from the 50 mM buffer) at 37 °C for 45 min. In the standard workflow, the digestion is terminated by diluting the digested samples (50 µL total volume) with 950 µL 0.1% formic acid (FA) in water. A total time of 45 min is required for sample preparation, and these samples are directly analyzed by the gradient LC–MS/MS method.

An SPE step added to provide a cleaner sample extract requires an additional 10 min. Here, 200 µL is taken from the abovementioned diluted digested samples, mixed with 700 µL of 0.1% formic acid in water and 100 µL of magnetic beads at 50 mg/mL, and purified by SPE by washing twice with 1 mL 0.1% FA in water and eluting with 1 mL 10% ACN and 0.1% formic acid in water. This step increases sample purity for the isocratic LC and FIA LC–MS/MS methods.

### LC–MS/MS methods

All LC methods described in Table 1 were developed on a Thermo Vanquish HPLC system modified to minimize extra-column volume, used 0.1% FA in water and 0.1% FA in acetonitrile (ACN) as LC solvents A and B, respectively, and shared a common MS method.

#### Gradient LC method with a run time of 36 s

The Waters 2.1 × 50 mm LC column (BEH C18 300 Å, 3.5-µm particles) was operated at a flow rate of 900 µL/min. After injection of 5 µL sample, the LC solvent composition was held at 15% eluent B between 0.00 and 0.30 min, ramped up from 15 to 70% between 0.30 and 0.35 min, held at 70% until 0.44 min, ramped down to 15% between 0.44 and 0.45 min, and held at 15% until 0.6 min. The total time between injections was about 0.9 min, which provided about 0.45 min for LC column equilibration. The 36-s LC gradient is illustrated in Supplementary Fig. 5.

#### Isocratic LC method with a run time of 12 s

The Waters Symmetry 2.1 × 10 mm Sentry Guard LC pre-column cartridge (C18 300 Å, 3.5-µm particles) was operated at a flow rate of 900 µL/min with a fixed LC solvent composition set to 10% eluent B and injection of 1 µL sample. The total time between injections was about 0.9 min.

#### FIA method with a run time of 18 s

FIA was performed using only an in-line filter at a flow rate of 150 µL/min with a fixed LC solvent composition set to 0% eluent B and injection of 1 µL sample. The total time between injections was about 0.9 min.

#### Thermo TSQ Quantiva parameters

The heated electrospray (H-ESI) source was coupled to the Thermo Vanquish HPLC system and operated at + 3.5 kV with the ion transfer tube temperature set to 350 °C and vaporizer temperature set to 400 °C, and the sheath gas, aux gas, and sweep gas were set to 50, 25, and 3, respectively. The doubly protonated-N-terminal hexapeptides were monitored at m/z 348.3 to 237.0 (non-glycated) with a collision energy of 22.9 and m/z 429.3 to 245.1 (glycated) with a collision energy of 16.5. MS resolution was set to 1.2 FWHM for both Q1 and Q3, and dwell time was set to 50 ms for both transitions.

### Data analysis

All raw data was processed (e.g., peak integration) using standard workflows available directly on the LC–MS system. The method is calibrated identically to the IFCC reference method, in short, by (1) measuring the HbA_1c_ and HbA_0_ signals for calibrators and calculating a linear fit of the ratio of these two signals and their molar ratios, (2) using the linear equation to determine the molar ratios of unknown samples, and (3) calculating the IFCC HbA_1c_ value using the relationship (molar ratio × 1000)/(1 + molar ratio).

## Results and discussion

### Development and evaluation of LC–MS/MS methods

The original IFCC reference method uses trifluoroacetic acid (TFA) as an ion-pairing agent in the LC solvents and an LC column with a cyanopropyl stationary phase to provide sufficient resolution of the HbA_0_ and HbA_1c_ peptides prior to targeted detection using positive-mode ESI and selected ion monitoring (SIM). We developed and optimized three different LC methods on the Thermo Vanquish HPLC system for quantitative determination of HbA_1c_ from human whole blood samples to explore (1) how much the LC methods could be accelerated by employing short LC columns containing small ID particles, operating at high flow rates, and a gradient optimized to resolve the HbA_1c_ and HbA_0_ peptides from other sample matrix components and (2) how much the demands on the LC methods could be reduced by transferring a portion of the separation to magnetic bead-based SPE. All of the optimized LC methods in this work used 0.1% FA in water and 0.1% FA in ACN as solvents A and B, respectively. Furthermore, because targeted detection using positive-mode ESI and selected reaction monitoring (SRM) was used in our methods, the selectivity is enhanced relative to SIM, and stable and accurate determination of co-eluting HbA_0_ and HbA_1c_ peptides during fast gradient or isocratic chromatography and during FIA could be achieved. Moreover, gradual signal loss from ion suppression and contamination of MS components common for LC methods using TFA could be avoided.

**Fig. 1:**
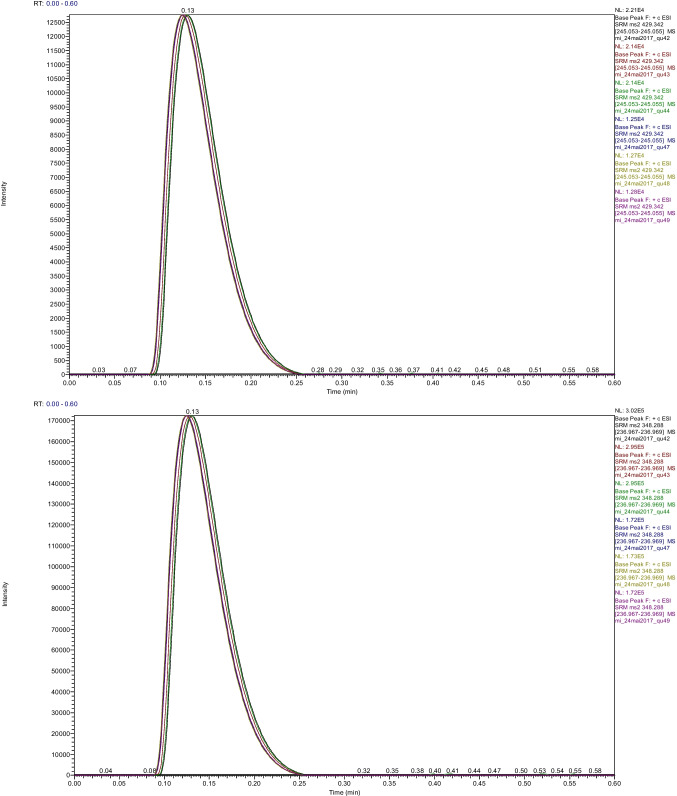
Overlaid extracted ion chromatograms of the SRM transitions of HbA_1c_ (top) and HbA_0_ (bottom) peptides from six replicate injections from a single whole blood sample digested with Glu C analyzed by the 36-s gradient LC–MS method

Figure 1 shows an overlay of chromatograms from six replicate injections from a single whole blood sample digested with Glu C and analyzed by the gradient LC method with a run time of 36 s. The analytes of interest eluted during the initial portion of the method as the LC solvent composition was held at 15% eluent B between 0.00 and 0.30 min and are thereby efficiently separated from sample matrix components that are retained by the LC column during MS/MS detection. The gradient was ramped up from 15 to 70% between 0.30 and 0.35 min and held at 70% until 0.44 min to clean the LC column and flow path and thereby minimize carryover. The gradient was ramped back down to 15% between 0.44 and 0.45 min and held at 15% until 0.6 min, and the total time between injections was about 0.9 min, which provided about 0.45 min to equilibrate the LC column and provide stable and reproducible MS detection. Using the same MS method, LC column, solvents, and a longer gradient that separates the HbA_0_ and HbA_1c_ peptides, we observed negligible HbA_0_ signal at the retention time of HbA_1c_ (data not shown). This result ensured negligible cross-analyte interference from in-source HbA_1c_ deglycosylation for the LC methods in this work, where the analytes are not chromatography resolved.

An SPE method using magnetic beads, which is described in the next section of this manuscript, provides a cleaner sample extract for the isocratic LC and FIA methods described below. Supplementary Fig. 2 shows chromatograms and overlay of three replicate injections from a single whole blood sample digested with Glu C and analyzed by the isocratic LC method with a run time of 12 s. As the analytes of interest eluted in a timeframe less than 0.20 min for the isocratic LC method, sample injection can theoretically occur every 12 s with a sufficiently fast LC injector. Supplementary Fig. 3 shows chromatograms and overlay of six replicate injections from a single whole blood sample digested with Glu C and analyzed by the FIA method with a run time of 18 s. As the analytes of interest eluted in a timeframe less than 0.30 min for the FIA method, sample injection can theoretically occur every 18 s with a sufficiently fast LC injector. The isocratic LC and FIA methods suggest that both cost and measurement time can be reduced by enriching analytes against sample matrix interferences through the use of magnetic bead-based SPE and employing a guard column or an in-line filter only instead of an LC column but were not characterized in detail.

### Development and evaluation of sample preparation methods

Table 1 compares the IFCC reference method to the method developed in this work. The IFCC reference method requires 5 h for sample pretreatment. In contrast, the method developed in this work performs sample pretreatment within 2 min by directly hemolyzing and diluting whole blood samples in a single step.

To optimize Glu C digestion speed and reproducibility, we screened Glu C concentration, Glu C purchased from different vendors and with different grades, temperature, time, agitation and mixing speeds, acetonitrile concentration, buffer concentration, and buffer salt and pH. Moreover, we also tried filter-aided digestion and screened denaturation strategies by elevated temperatures and various denaturants (e.g., SDS, guanidine HCl, Urea, Protease MAX surfactant, Rapigest surfactant, PPS surfactant, DOC surfactant) and screened digestion enhancers including Dodecyl-β-D-maltoside and Trehalose. Ultimately, we found that the fastest, simplest, and most reproducible conditions are when 5-µL samples are digested with 0.6 mg/mL Glu C in 30 mM ammonium acetate buffer (pH 4.3) at 37 °C and the digestion is terminated by diluting the samples in 0.1% formic acid in water. The Glu C digestion kinetics of our method is compared to the IFCC reference method in Supplementary Fig. 4 and produces nearly 80 × higher signal after 30-min incubation due to the 30 × higher Glu C concentration and optimized (lower) buffer concentration.

**Fig. 2:**
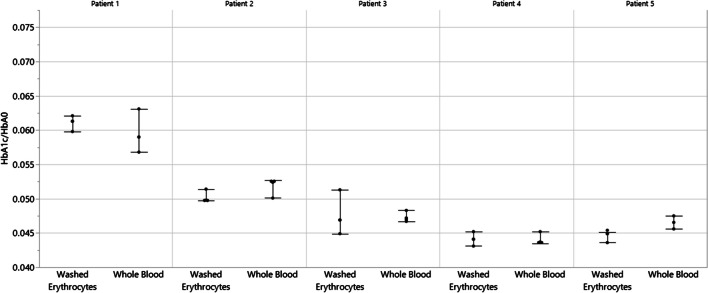
Ratio of HbA_1c_ and HbA_0_ determined for five different patient samples comparing two different pretreatment methods. The pretreatment method from the IFCC reference method extracts erythrocytes (red blood cells) from whole blood samples and destroys labile HbA_1c_ by washing and incubation with a saline solution, releases hemoglobin (Hb) from the purified erythrocytes by hemolysis using deionized water, and adjusts the Hb concentration to 50 mg/mL by UV-based determination and dilution with a storage buffer. In contrast, the pretreatment method optimized in this work involves direct hemolysis and dilution of whole blood samples with deionized water prior to optimized digestion using Glu C. The mean HbA_1c_/HbA_0_ ratio and standard deviation are displayed from three sample preparation replicates and single LC–MS determinations of all five patient samples using both pretreatment methods

**Fig. 3:**
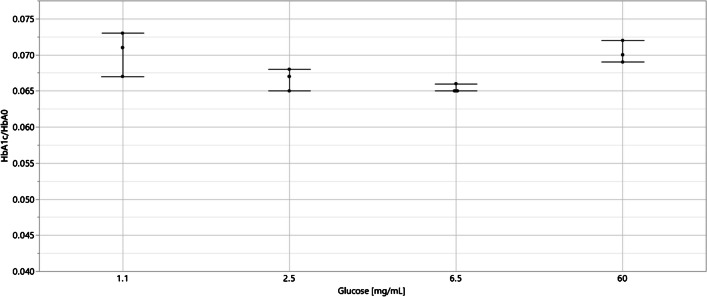
Ratio of HbA_1c_ and HbA_0_ determined for a single patient sample incubated in various concentrations of glucose to detect the presence of labile HbA_1c_. The whole blood patient samples in 1 mL amounts were gently mixed with dry glucose (1.1 mg, 2.5 mg, 6.5 mg, and 60 mg) and incubated at 37 °C for 3 h prior to optimized hemolysis and enzymatic digestion method using Glu C. Extra care was taken to ensure gentle mixing of the whole blood samples with glucose by using a roller to prevent hemolysis. The mean HbA_1c_/HbA_0_ ratio and standard deviation are displayed from three sample preparation replicates and single LC–MS determinations

**Fig. 4:**
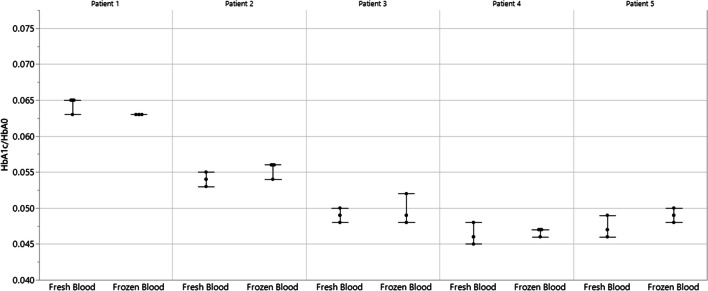
Ratio of HbA_1c_ and HbA_0_ determined for five different patient samples comparing fresh and frozen samples. The mean HbA_1c_/HbA_0_ ratio and standard deviation are displayed from three sample preparation replicates and single LC–MS determinations of all five patient samples that were either freshly prepared or frozen

The following results ensure that the modifications of the current sample preparation method relative to the IFCC reference method did not introduce any problems in HbA_1c_ determination. Figure 2 plots the ratio of HbA_1c_ and HbA_0_ determined for five different patient samples prepared by the pretreatment methods of the IFCC reference methods and the current method and demonstrates that HbA_1c_ determination is not affected. Figure 3 plots the ratio of HbA_1c_ and HbA_0_ determined for a single patient sample incubated in various concentrations of glucose (1.1 mg/mL, 2.5 mg/mL, 6.5 mg/mL, and 60 mg/mL) and demonstrates that the current method does not detect labile HbA_1c_ and, thus, the 4-h incubation used in the IFCC reference method could be safely omitted. Figure 4 plots the ratio of HbA_1c_ and HbA_0_ determined for five different patient samples comparing fresh and frozen samples and demonstrates that the current method determined the same HbA_1c_ values comparing fresh and frozen samples, which is an important consideration for calibrator and QC materials and cross-referencing or re-running patient samples stored at − 20 °C or lower temperatures.

The SPE method using magnetic beads provided a cleaner sample extract for the isocratic LC and FIA methods by minimizing sample matrix components that elute with stronger LC solvent composition. Elution with 10% ACN allows selective elution of HbA_1c_ and HbA_0_ peptides prior to LC–MS. This purification step provided stable and reproducible MS detection of a lower amount of sample and allowed isocratic conditions by alleviating the need to clean the pre-column with stronger solvents. Supplementary Fig. 1 shows the result from digestion of whole blood samples with Glu C without further purification (top) and with sample purification by enrichment on magnetic beads, followed by washing and elution with different concentrations of ACN (i.e., 5%, 10%, 15%, 20%, and 30%).

Although we tested and identified other proteolytic enzymes that are faster, either naturally or due to genetic or chemical modifications, and explored a method to determine the intact β-chain of Hb, we focused on Glu C in this work because the N-terminal hexapeptides are largely conserved in human genetics, at least in heterozygous form, and can be reliably analyzed by LC–MS methods and for comparability with the established IFCC reference method. Lastly, we do not recommend elevated temperatures at any point during sample preparation because we observed that HbA_1c_ signal was reduced, presumably due to thermal degradation (data not shown).

### Evaluation of method linearity, precision, and accuracy

**Fig. 5:**
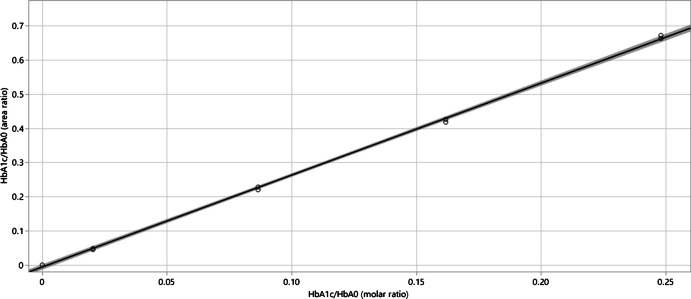
Linearity of 0, 20, 80, 140, and 200 HbA_1c_/mol Hb (IFCC) prepared from purified glycated and non-glycated hemoglobin reference materials and analyzed by the developed method cover and extend well beyond the physiologically relevant HbA_1c_ range and correspond to 0.0%, 4.0%, 9.4%, 15%, and slightly over 20% HbA_1c_ NGSP units, respectively. The correlation coefficient is 0.999

As shown in Fig. 5, the ratio of HbA_1c_ and HbA_0_ signals (i.e., method calibration curve) is linear across the entire range of working calibrators prepared for this study with a correlation coefficient of 0.999. Specifically, the 0, 20, 80, 140, and 200 HbA_1c_/mol Hb (IFCC) made from purified glycated and non-glycated hemoglobin reference materials cover and extend well beyond the physiologically relevant HbA_1c_ range and correspond to 0.0%, 4.0%, 9.4%, 15%, and slightly over 20% HbA_1c_ NGSP units, respectively.

Mean values and imprecision of determined HbA_1c_ (IFCC mmol/mol) by the developed method of two sample preparation replicates and single LC–MS determinations over 10 days (*n* = 20) for 15 different patient samples are listed in Table 2. The range of reproducibility is between 1.3 and 2.3%.

**Table 2:** Mean values and imprecision of determined HbA_1c_ (IFCC mmol/mol) by the developed method of two sample preparation replicates and single LC–MS determinations over 10 days (*n* = 20) for 15 different patient samples

Patient	HbA_1c_ (IFCC mmol/mol)	CV (%)
1	46.9	1.7%
2	29.7	2.0%
3	47.1	1.3%
4	47.7	2.0%
5	37.8	2.3%
6	42.7	1.7%
7	41.8	1.8%
8	73.8	1.8%
9	60.3	1.4%
10	25.2	2.1%
11	25.9	2.2%
12	31.6	2.1%
13	24.8	1.7%
14	27.0	1.7%
15	29.1	2.1%

**Fig. 6:**
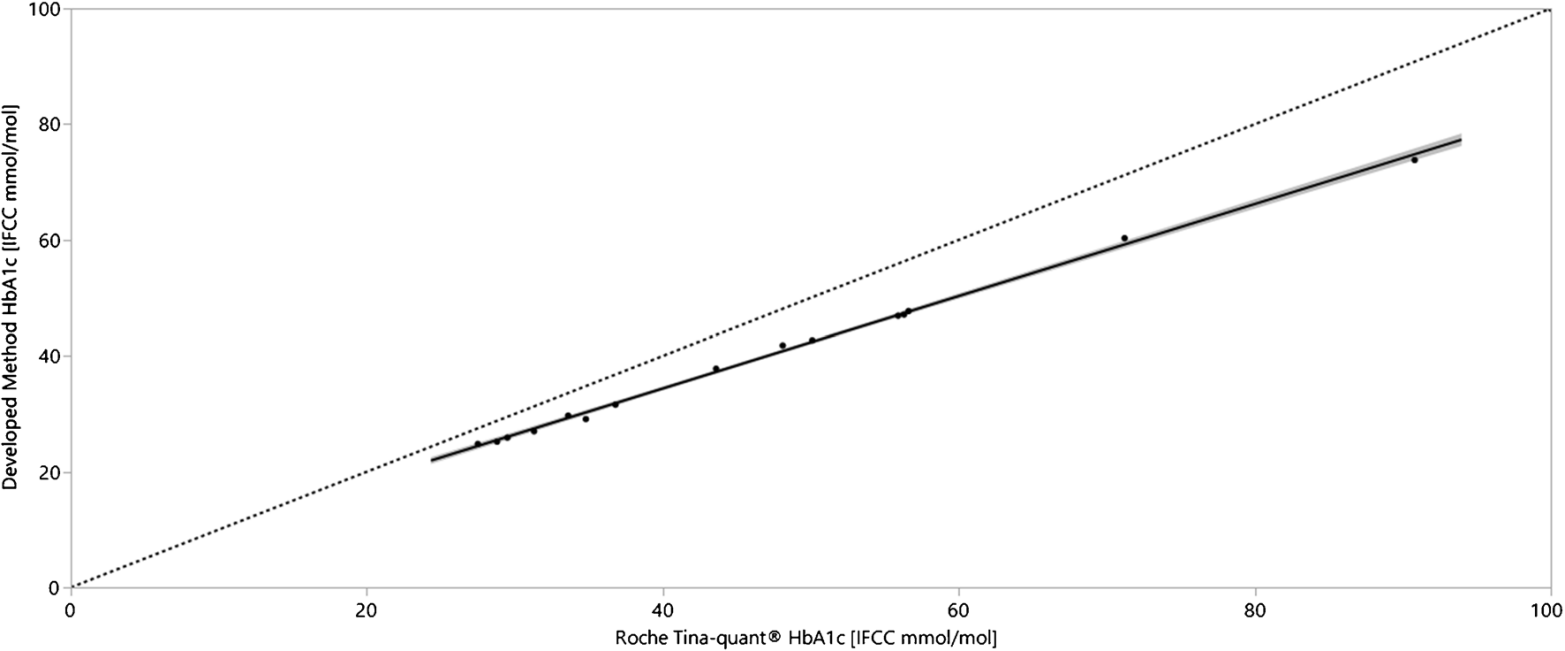
Measurement range and correlation of determined HbA_1c_ (IFCC mmol/mol) from the c 513 system running the Tina-quant HbA_1c_ Gen. 3 assay and from the developed method for 15 different patient samples. The mean values from two sample preparation replicates and single LC–MS determinations over 10 days (*n* = 20) are compared to three sample preparation replicates and single determinations from the c 513 system running the Tina-quant HbA_1c_ Gen. 3 assay (*n* = 3). The correlation coefficient is 0.998 but the HbA_1c_ value is underestimated between 10 and 19% across the HbA_1c_ range of the patient samples

The measurement range and correlation of determined HbA_1c_ (IFCC mmol/mol) from the Roche Cobas® c 513 system running the Cobas Tina-quant® HbA_1c_ Gen. 3 assay and from the current method for 15 different patient samples are shown in Fig. 6. The mean values from two sample preparation replicates and single determinations by LC–MS over 10 days (*n* = 20) compared to three sample preparation replicates and single determinations from the c 513 system running the Tina-quant HbA_1c_ Gen. 3 assay (*n* = 3) with a correlation coefficient of 0.998. Albeit this strong correlation, the method developed in this work underestimated the HbA_1c_ value between 10 and 19% across the HbA_1c_ range of the patient samples.

Although absolute standardization and method validation were not the focus of our feasibility study, considerations about patient safety and method accuracy are essential and further efforts are required to identify the source of the bias and correct it. Groups interested in continuing this work might consider (1) verification and correction of the calibrator materials by comparing to certified reference samples and ensuring their purity and stability through robust standardization and formulation, (2) using heavy isotope-labeled peptide ISTDs, (3) performing magnetic bead-based SPE prior to the gradient LC–MS method, (4) further optimizations of the sample preparation and LC–MS/MS methods, and (5) a comprehensive method validation.

## Conclusion

In conclusion, we developed and demonstrated feasibility of methods for HbA_1c_ determination that require less than 1 h to prepare whole blood samples (fast hemolysis, enzymatic digestion, and optional magnetic bead-based SPE) and 36 s, 18 s, and 12 s for LC–MS/MS and FIA-MS/MS detection with an analytical performance comparable to the IFCC reference method. These short times for sample preparation and analysis and stringent levels of reproducibility, accuracy, and simplicity are necessary for routine clinical settings. Lastly, this work contributes to the greater body of literature demonstrating the feasibility of fast and reliable clinically relevant LC–MS-based protein quantification.

## Supplementary Information

Below is the link to the electronic supplementary material.

Supplementary file1 (DOCX 347 KB)

## Declarations

### Source of biological material

Whole blood samples were donated by anonymous volunteers at Roche Diagnostics GmbH (Penzberg, Germany).
