# Supplementary material for: Early developments toward HbA1c determination in whole blood by high-speed sample preparation and LC–MS/MS analysis
Source: Anal Bioanal Chem. 2024 Oct 26;416(29):6735–44. doi: 10.1007/s00216-024-05601-5 (PMC11579156; doi:10.1007/s00216-024-05601-5)
Supplement: Supplementary file 1 — Supplementary file1 (DOCX 347 KB) [file 216_2024_5601_MOESM1_ESM.docx]

**Supplementary Figure 1:** Chromatograms from bead washing experiments with different elution conditions. These experiments were conducted using a Q Exactive MS system with a long LC-MS method to resolve HbA_1c_ and HbA_0_ peaks and detect other sample components.

**Supplementary Figure 2:** Overlaid extracted ion chromatograms of the SRM transitions of HbA_1c_ (top) and HbA_0_ (bottom) peptides from three replicate injections from a single whole blood sample digested with Glu C analyzed by the 12 s isocratic LC-MS method.


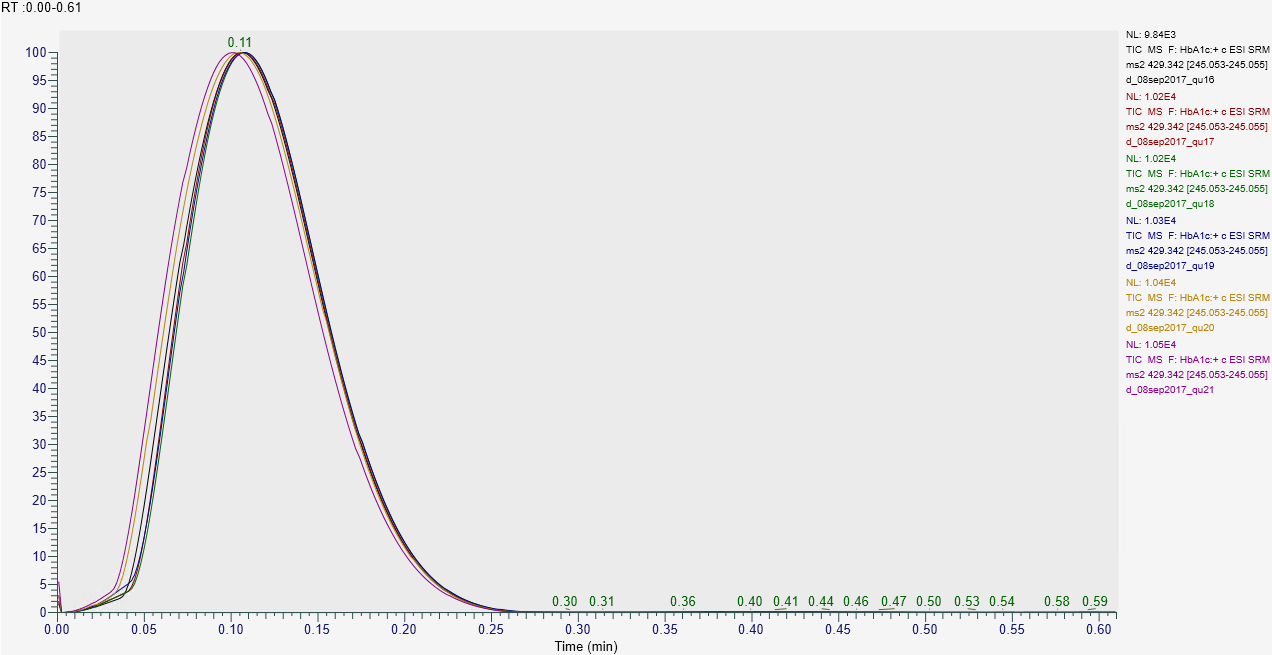


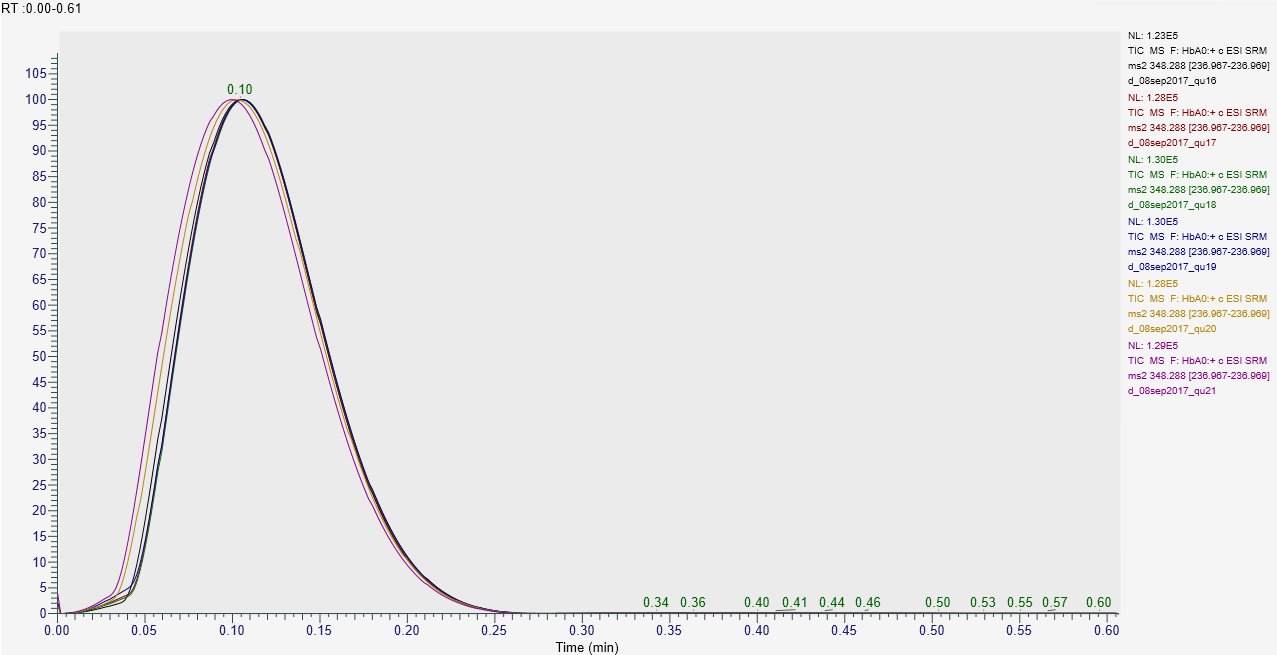


**Supplementary Figure 3:** Overlaid extracted ion chromatograms of the SRM transitions of HbA_1c_ (top) and HbA_0_ (bottom) peptides from six replicate injections from a single whole blood sample digested with Glu C analyzed by the 18 s FIA method.


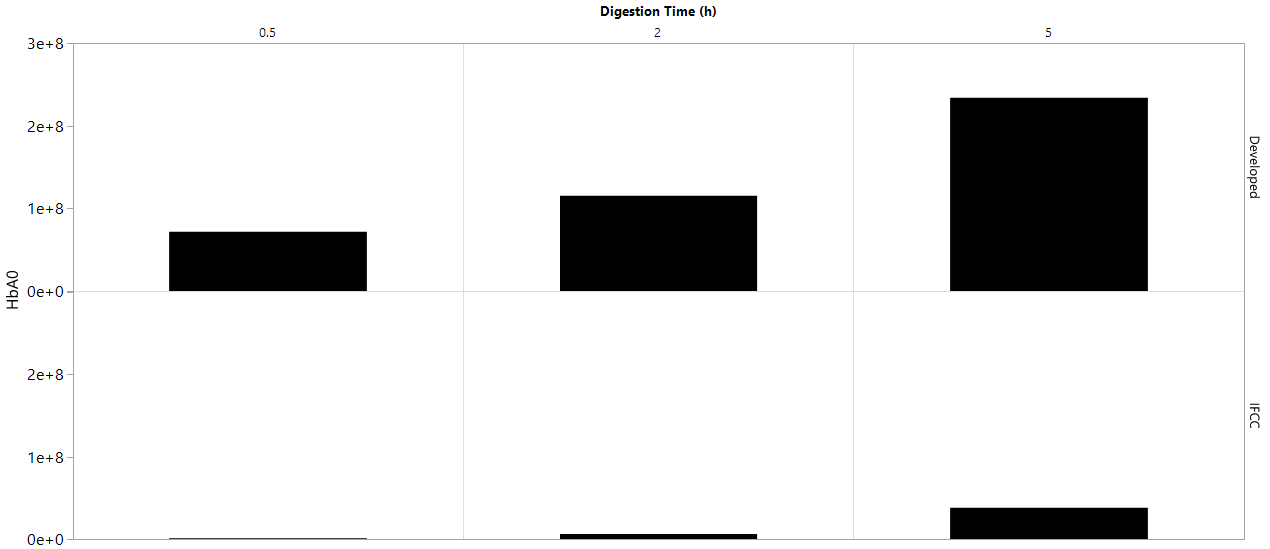


**Supplementary Figure 4:** HbA_0_ signal as a function of time comparing two different Glu C digestion methods modified to filter-aided digestion for this kinetics experiment. The method developed in this work produces nearly 80x higher signal after 30 min incubation time compared to the IFCC reference method due to the 30x higher Glu C concentration and optimized (lower) buffer concentration, as listed in Table 1. These experiments were conducted using a Q Exactive MS system with a long LC-MS method to resolve HbA_1c_ and HbA_0_ peaks and detect other sample components.


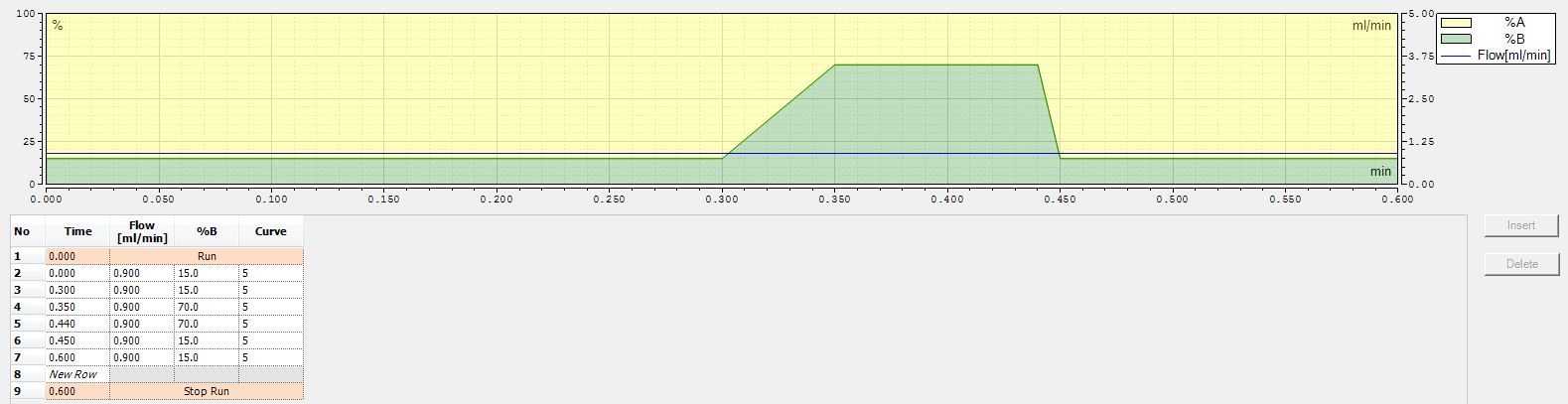


**Supplementary Figure 5:** Gradient LC method on the Thermo Vanquish HPLC system with run time of 36 s: The Waters 2.1 x 50 mm LC column (BEH C18 300 Å, 3.5 µm particles) was operated at flow rate of 900 µL/min. After injection of 5 µL sample, the LC solvent composition was held at 15% eluent B between 0.00 and 0.30 min, ramped up from 15% to 70% between 0.30 and 0.35 min, held at 70% until 0.44 min, ramped down to 15% between 0.44 and 0.45 min, and held at 15% until 0.6 min. The total time between injections was about 0.9 min, which provided about 0.45 min for LC column equilibration.

**Supplementary Table 1:** Calibrators prepared manually from purified glycated and non-glycated Hb that are produced in house at Roche Diagnostics GmbH, Penzberg and typically used to prepare candidate primary reference material approved by the IFCC.

| Material | Mol. Wt. (g/mol) | Stock (mg/mL) | Stock (M) | HbA1c / HbA0 | IFCC HbA1c (mmol HbA1c / mol Hb) | HbA0 (µL) | HbA1c (µL) | Storage Buffer (µL) | Total Volume (µL) |
| --- | --- | --- | --- | --- | --- | --- | --- | --- | --- |
| HbA0 Protein | 15867.2 | 163.0 | 1.03E-02 |  | --- | --- | --- | --- | --- |
| HbA1c Protein | 16029.4 | 33.5 | 2.09E-03 |  | --- | --- | --- | --- | --- |
|  |  |  |  |  |  |  |  |  |  |
|  |  | Total Hb (mg/mL) |  |  |  |  |  |  |  |
|  |  | 23.8 |  | 0.000000 | 0.000 | 51 | 0 | 299 | 350 |
|  |  | 23.8 |  | 0.020344 | 19.939 | 50 | 5 | 295 | 350 |
|  |  | 23.8 |  | 0.086571 | 79.674 | 47 | 20 | 283 | 350 |
|  |  | 23.8 |  | 0.161829 | 139.288 | 44 | 35 | 271 | 350 |
|  |  | 23.9 |  | 0.248100 | 198.782 | 41 | 50 | 259 | 350 |
|  |  |  |  |  |  |  |  |  |  |
| Note: Calculations based on normal (nonvariant) human hemoglobin | | | | | | | | | |
